# C/EBP homologous protein (CHOP) deficiency ameliorates renal fibrosis in unilateral ureteral obstructive kidney disease

**DOI:** 10.18632/oncotarget.7870

**Published:** 2016-03-03

**Authors:** Shing-Hwa Liu, Cheng-Tien Wu, Kuo-How Huang, Ching-Chia Wang, Siao-Syun Guan, Li-Ping Chen, Chih-Kang Chiang

**Affiliations:** ^1^ Institute of Toxicology, College of Medicine, National Taiwan University, Taipei, Taiwan; ^2^ Department of Pediatrics, College of Medicine, National Taiwan University & Hospital, Taipei, Taiwan; ^3^ Department of Medical Research, China Medical University Hospital, China Medical University, Taichung, Taiwan; ^4^ Department of Urology, College of Medicine, National Taiwan University, Taipei, Taiwan; ^5^ Institute of Nuclear Energy Research, Atomic Energy Council, Taoyuan, Taiwan; ^6^ Department of Dentistry, Taipei Chang Gang Memorial Hospital, Chang Gang University, Taipei, Taiwan; ^7^ Department of Integrated Diagnostics & Therapeutics, National Taiwan University Hospital, Taipei, Taiwan

**Keywords:** CHOP, renal fibrosis, unilateral ureteral obstruction, oxidative stress

## Abstract

Renal tubulointerstitial fibrosis is an important pathogenic feature in chronic kidney disease and end-stage renal disease, regardless of the initiating insults. A recent study has shown that CCAAT/enhancer binding protein (C/EBP) homologous protein (CHOP) is involved in acute ischemia/reperfusion-related acute kidney injury through oxidative stress induction. However, the influence of CHOP on chronic kidney disease-correlated renal fibrosis remains unclear. Here, we investigated the role of CHOP in unilateral ureteral obstruction (UUO)-induced experimental chronic tubulointerstital fibrosis. The CHOP knockout and wild type mice with or without UUO were used. The results showed that the increased expressions of renal fibrosis markers collagen I, fibronectin, α-smooth muscle actin, and plasminogen activator inhibitor-1 in the kidneys of UUO-treated wild type mice were dramatically attenuated in the kidneys of UUO-treated CHOP knockout mice. CHOP deficiency could also ameliorate lipid peroxidation and endogenous antioxidant enzymes depletion, tubular apoptosis, and inflammatory cells infiltration in the UUO kidneys. These results suggest that CHOP deficiency not only attenuates apoptotic death and oxidative stress in experimental renal fibrosis, but also reduces local inflammation, leading to diminish UUO-induced renal fibrosis. Our findings support that CHOP may be an important signaling molecule in the progression of chronic kidney disease.

## INTRODUCTION

Renal tubulointerstitial fibrosis is an important hallmark during the progression from chronic kidney disease (CKD) to the end-stage renal disease (ESRD). Fibrosis is the final common pathway of the majority of chronic renal diseases regardless of the initiating insult [1]. Accumulating evidence indicates that tubulointerstitial injury is a more consistent predictor of renal functional decline than glomerular injury [2, 3]. Renal tubulointerstitial fibrosis reflects the imbalance of different mechanisms including renal cells apoptosis, inflammatory cells infiltration, and oxidative stress generation. However, the molecular mechanisms of renal tubulointerstitial fibrosis are still not well understood. Studies aiming to elucidate the potential mechanisms of renal fibrosis are urgently needed to facilitate the discovery of therapies capable of reversing renal fibrosis and improving CKD and ESRD. Endoplasmic reticulum (ER) stress has been shown to be triggered by cellular insults including starvation, genetic mutation, disturbance of protein turnover, and inflammation, and may be associated with diseases such as diabetes, cardiomyopathy, and neuron degeneration disease [4–8]. Furthermore, several studies identified that ER stress might take part in the process of cholestasis-induced liver fibrosis, cystic fibrosis, pulmonary fibrosis, and renal fibrosis [4, 9–11].

The CCAAT/enhancer binding protein (C/EBP) homologous protein (CHOP), also known as C/EBPβ DNA damage-inducible gene 153 (GADD153), or DNA damage inducible transcript 3 (DDIT3), is an important transcription factor that contributes to numerous cellular functions such as apoptosis, inflammation, and differentiation [12–15]. CHOP is one of highest inducible genes during ER stress. In several animal disease models like as diabetes, neuron degeneration, and ischemia/reperfusion in brain or kidney, CHOP is involved in cellular apoptosis and organ dysfunction [16–19]. CHOP has also been found to participate in PGE_2_-stimulated IL-8 production in cystic fibrosis bronchial epithelial cells [9], the regulation of cell-matrix adhesion in podocytes [20], and oxidative stress-related apoptosis in neuroblastoma cells [21]. CHOP can induce reactive oxygen species (ROS) formation and inflammation during acute renal ischemia-reperfusion (I/R) injury [22]. It is expected that prolonged ROS generation and inflammation in acute renal injury may also contribute to renal fibrosis chronically. However, the role of CHOP activation during ER stress in obstruction-induced renal fibrosis is still unclear. To address this issue, we hypothesized that ER stress-related CHOP is involved in the pathology of chronic renal fibrosis. In this study, *CHOP*-knockout mice subjected to unilateral ureteral obstruction (UUO), a conventional approach for inducing renal fibrosis, resulting in accumulation of extracellular matrix, prolonged oxidative stress, and acceleration of interstitial inflammation [23–25], were used. We demonstrated that CHOP deficiency effectively ameliorated obstruction-induced renal fibrosis via the attenuation of profibrotic factors, oxidative stress, and inflammatory cells infiltration.

## RESULTS

### CHOP deficiency attenuated renal fibrosis in a mouse UUO model

**Figure 1:**
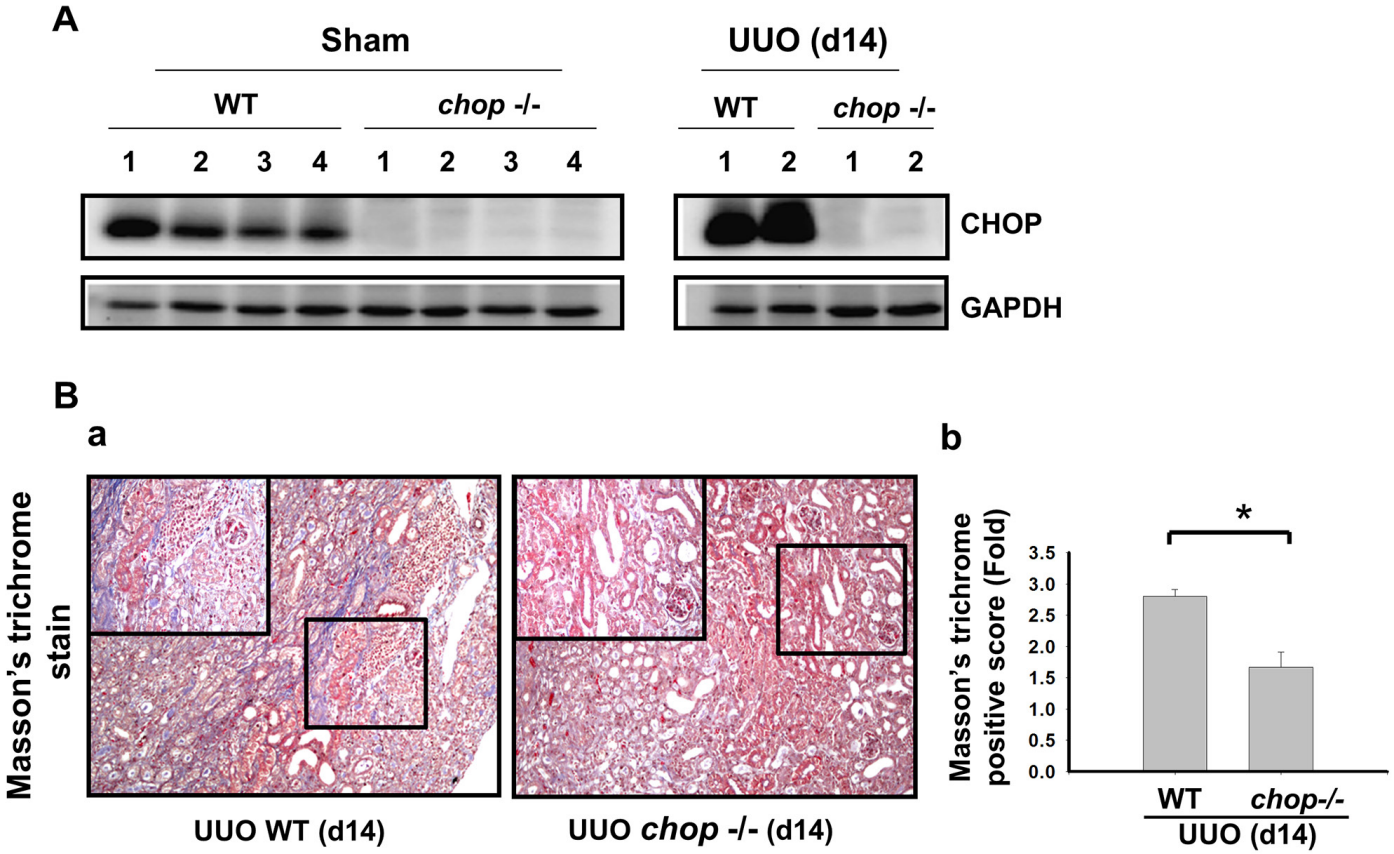
CHOP deficiency alleviates UUO-induced collagen deposition in fibrotic kidney Wild type or *CHOP* knockout C57BL/6 mice were surgically administered with UUO surgery for 14 days. CHOP protein expression were assessed by Western blotting assay **A.** Results are representative of at least three independent experiments from four mice per group. Pathological changes of renal fibrosis in UUO treatment of wild type and *CHOP* deficiency mice were performed by the Masson's trichrome staining **B.** The quantification was shown in (B-b). Data are presented as mean ± SEM (n=4/group) for three independent experiments. *P < 0.05, UUO (day 14) wild type vs *CHOP*−/−. WT: wild type; *CHOP*−/−: CHOP deficiency.

**Figure 2:**
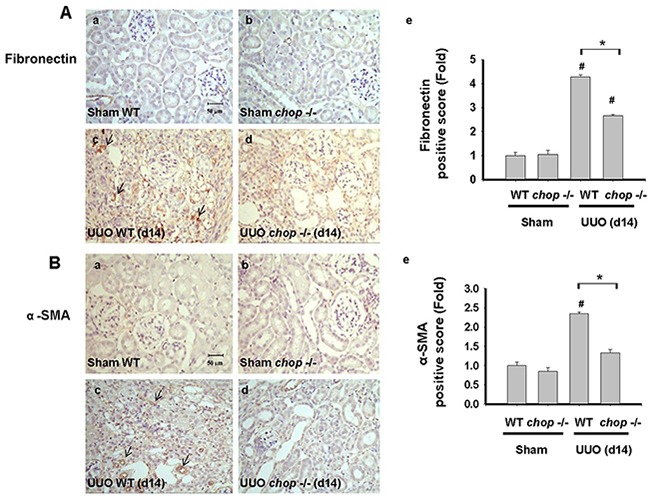
CHOP deficiency mitigates UUO-induced FN and α-SMA expression Wild type or *CHOP* knockout C57BL/6 micewere surgically administered with UUO surgery for 14 days. Sham control was the contralateral normal kidneys. Pathological changes of renal fibrosis were displayed by the IHC staining for fibronectin **A-a.** to **A-d.** and αSMA, **B-a.** to **B-d.** The quantification was showed in **B-e.** Data are presented as mean ± SEM (n=4/group) for three independent experiments. #*P* < 0.05, vs wild type sham control. *P <0.05, UUO (day 14) wild type vs *CHOP*−/−. WT: Wild type; *CHOP*−/−: CHOP deficiency; α -SMA: α-smooth muscle actin.

**Figure 3:**
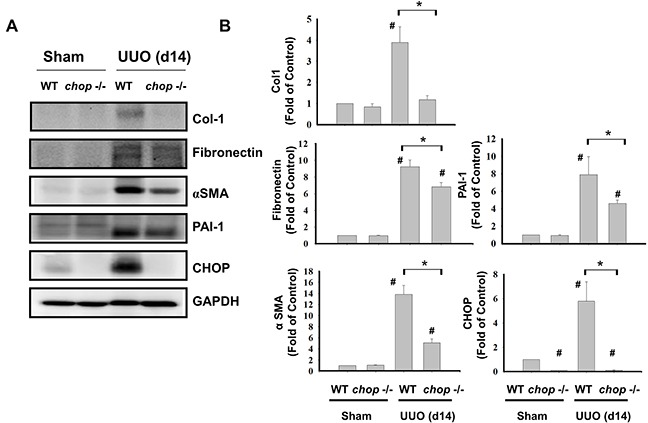
CHOP deficiency attenuates fibrosis-related markers in the kidneys of UUO mice Wild type or *CHOP* knockout C57BL/6 micewere surgically administered with UUO surgery for 14 days. Sham control was the contralateral normal kidneys. The expressions of renal fibrosis markers collagen I (Col-1), fibronectin, α-smooth muscle actin (αSMA), and plasminogen activator inhibitor-1 (PAI-1) and CHOP were performed by the Western blotting **A.** The quantification was shown in **B.** Data are presented as mean ± SEM (n=5/group) for three independent experiments. #*P* < 0.05, vs wild type sham control. *P <0.05, UUO (day 14) wild type vs *CHOP*−/−. WT: Wild type; *CHOP*−/−: CHOP deficiency.

As shown in Figure 1A, Western blotting displayed that CHOP expression was upregulated in the wild type (WT) mice with UUO on day 14. In KO mice, neither sham control nor the UUO kidney expressed CHOP on day 14 (Figure 1A). Moreover, Masson's Trichrome staining revealed that kidneys of WT mice possess obvious tubulointerstitial collagen deposition in response to UUO, while only mild collagen deposition is observed in the kidneys of KO mice with UUO (Figure 1B-a and 1B-b). As shown in Figure 2, the expressions of both fibronectin, a major ECM protein and a fibroblast chemoattractant [26], and αSMA, a tubulointerstitial fibrosis marker in UUO kidney [2], were barely detectable in the kidneys of sham WT or sham KO mice, but prominently elevated in the WT mice after UUO on day 14. On the contrary, both fibronectin and αSMA expressions were significantly decreased in the kidneys of KO mice after UUO on day 14, as compared with WT mice with UUO (Figures 2A and 2B). Similarly, Western blotting also showed that the increased expressions of collagen I, fibronectin, αSMA, and plasminogen activator inhibitor-1 (PAI-1) in UUO kidneys were significantly reversed in the *CHOP* KO mice (Figure 3). These results suggest that *CHOP* deficiency effectively restores the UUO-induced renal fibrosis.

### CHOP deficiency decreases renal cell apoptosis in UUO kidney

**Figure 4:**
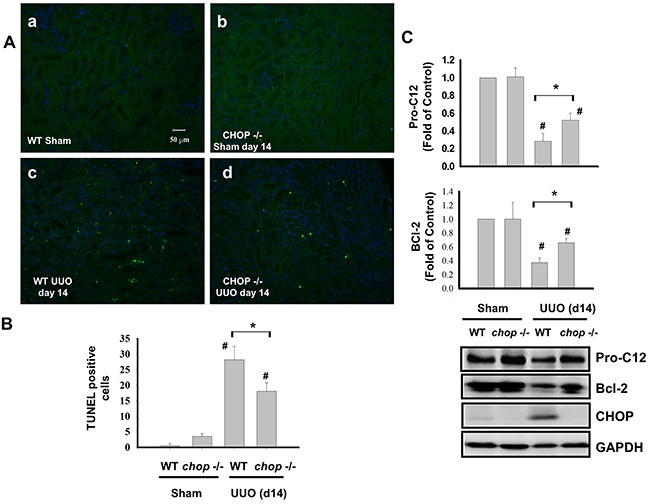
CHOP deficiency attenuates renal cell apoptosis in the kidneys of UUO mice Wild type or *CHOP* knockout C57BL/6 micewere surgically administered UUO for 14 days. Sham control was the contralateral normal kidneys. Apoptotic cells in renal were assessed by the TUNEL staining **A.** Quantification was counted by blinding selection for 10 random visual fields, using 200X magnification **B.** The expressions of apoptosis-related proteins Pro-caspase 12, Bcl-2, and CHOP were performed by the Western blotting **C.** Data are presented as mean ± SEM (n=5/group) for three independent experiments. #*P* < 0.05, vs wild type sham control. *P <0.05, UUO (day 14) wild type vs *CHOP*−/−. WT: Wild type; *CHOP*−/−: CHOP deficiency.

Tubular apoptosis is a critical feature of renal implications in the development of tubular functional atrophy or chronic progressive renal disease [24, 27, 28]. *CHOP* is known as an inducible gene after growth arrest, DNA damage and transcriptional regulator inducing the ER stress-correlated apoptosis [29]. As shown in Figure 4A and 4B, the increased apoptosis was obviously observed in the kidneys of WT mice with UUO on day 14, but not incited in KO mice. Furthermore, the increase of Pro-caspase 12 and Bcl-2 cleavage in the kidneys of WT mice with UUO obviously observed than in KO mice on day 14 (Figure 4C). These results indicated that CHOP deficiency decreases renal cell apoptosis in chronic renal fibrosis.

### CHOP deficiency abated reactive oxygen species (ROS) production in UUO kidney

**Figure 5:**
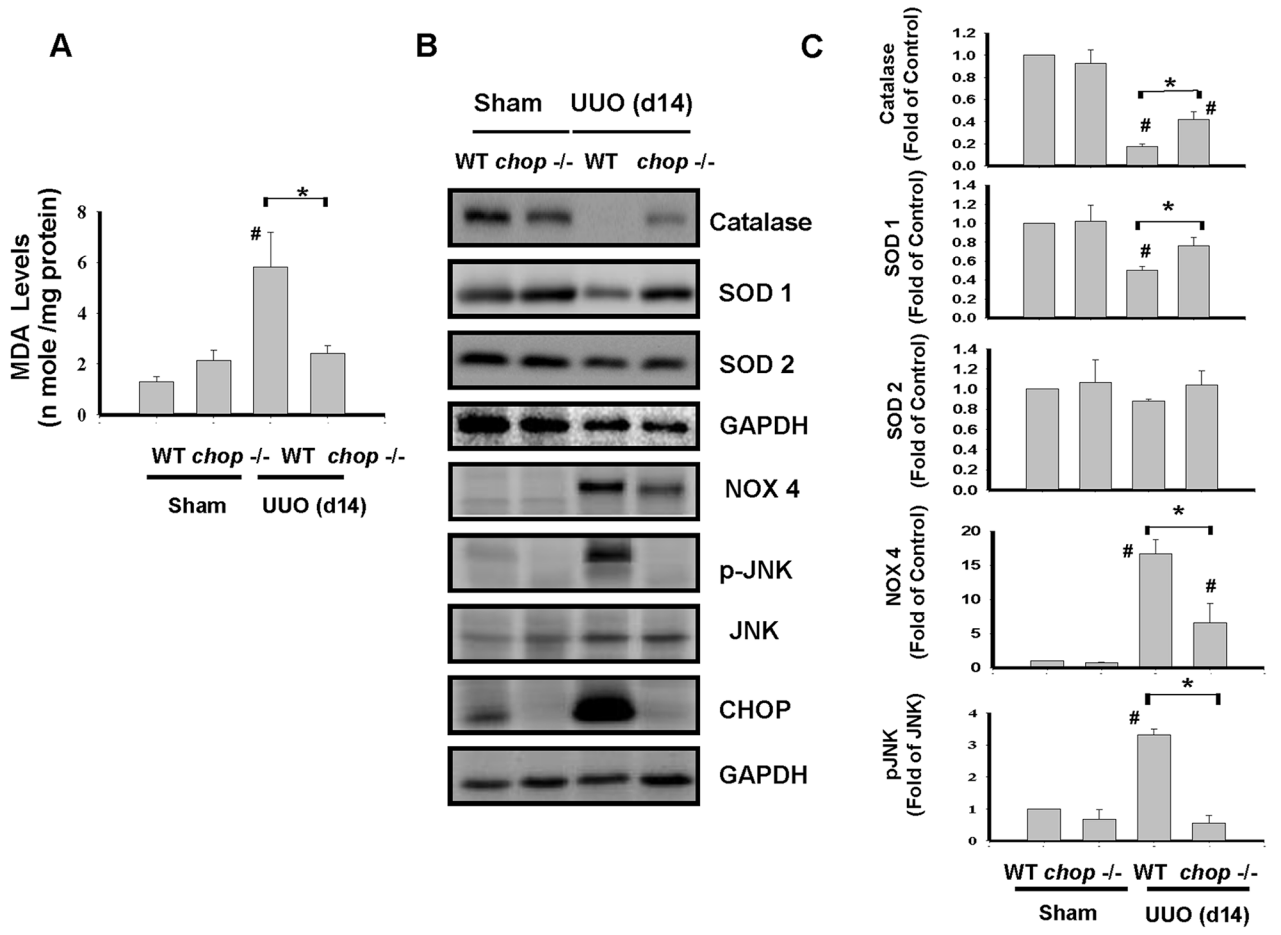
CHOP deficiency abates oxidative stress in the kidneys of UUO mice Wild type or *CHOP* knockout C57BL/6 micewere surgically administered UUO for 14 days. Sham control was the contralateral normal kidneys. Oxidative stress was assessed by the MDA stain in **A.** The expressions of oxidative-related proteins Catalase, superoxide dismutase 1 (SOD1), SOD2, NADPH oxidase 4 (NOX4), JNK and its phosphorylated form, and CHOP were performed by the Western blotting **B.** Quantification was shown in **C.** Data are presented as mean ± SEM (n=5/group) for three independent experiments. #*P* < 0.05, vs wild type sham control. *P <0.05, UUO (day 14) wild type vs *CHOP*−/−. WT: Wild type; *CHOP*−/−: CHOP deficiency.

ROS is known as a key mediator involved in the obstructive-induced renal fibrosis [30–32]. Our previous study indicated that CHOP may effectively prevent hypoxia/reperfusion-induced ROS injury. Consequently, we tested whether *CHOP* deficiency is involved in the attenuation of ROS production in chronic renal fibrosis. As shown in Figure 5A, the product of lipid peroxidation, malondialdehyde (MDA) was significantly increased in UUO kidneys of WT mice but not in KO mice on day 14. Moreover, NADPH oxidase isoform NOX-4 protein expression was increased, but superoxide dismutase (SOD1 and SOD2) and catalase protein expressions were decreased in UUO kidneys of WT mice, which are shown to counteract in *CHOP* deficiency mice (Figure 5B and 5C). These results indicated that *CHOP* deficiency can attenuate UUO-induced renal fibrosis via attenuating oxidative stress.

### CHOP deficiency diminished inflammation cells recruitment but did not activate NFκB or C/EBPβ expression in obstructive kidney

**Figure 6:**
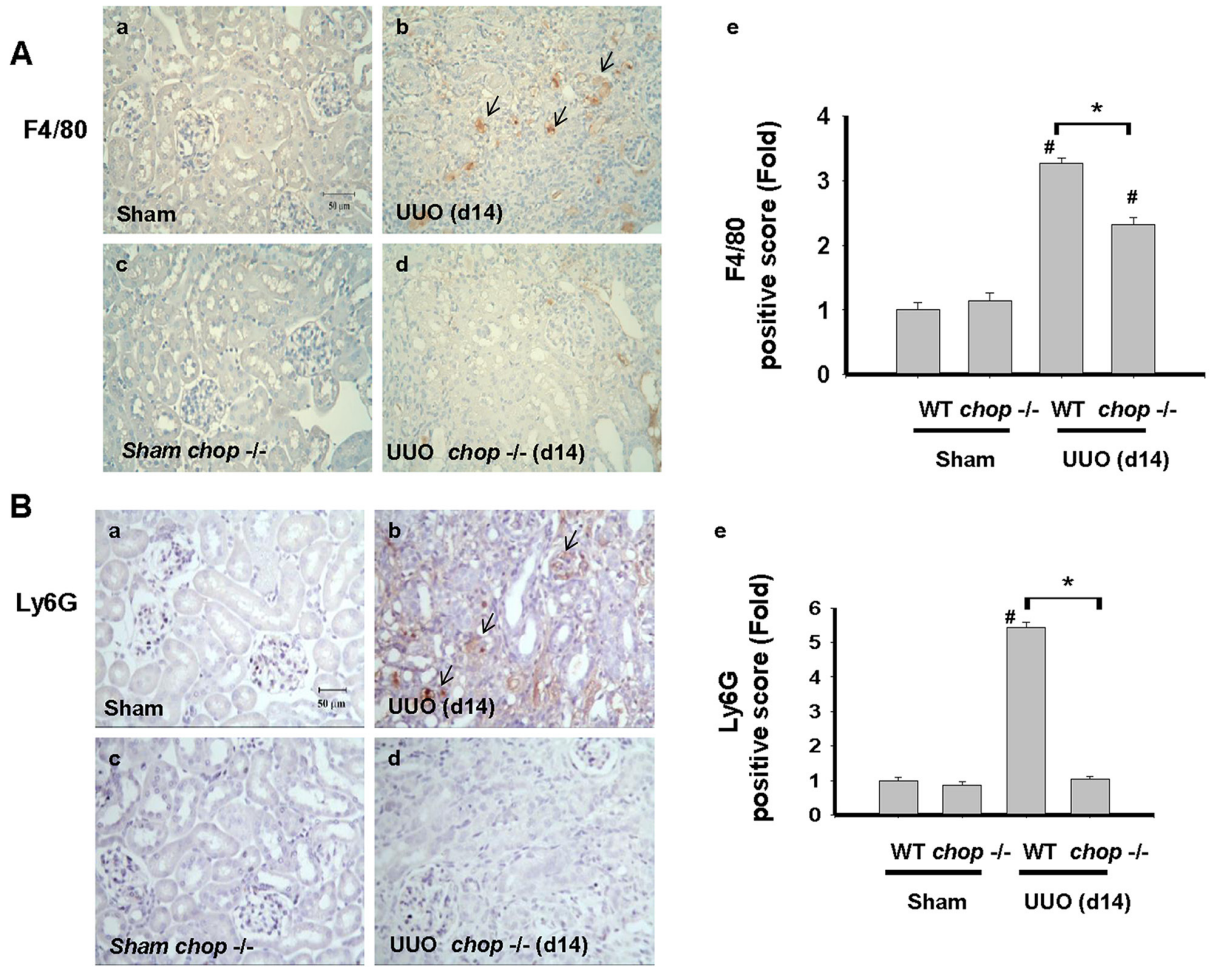
CHOP deficiency attenuates macrophages and neutrophils infiltration in the kidneys of UUO mice Wild type or *CHOP* knockout C57BL/6 micewere surgically administered UUO for 14 days. Sham control was the contralateral normal kidneys. The macrophage marker F4/80 was detected by the IHC staining and the quantification was detected in **A.** The neutrophil marker Ly6G was detected by the IHC staining and its quantification was shown in **B.** Data are presented as mean ± SEM (n=5/group) for three independent experiments. #*P* < 0.05, vs wild type sham control. *P <0.05, UUO (day 14) wild type vs *CHOP*−/−. WT: Wild type; *CHOP*−/−: CHOP deficiency.

Inflammation is another important feature, contributing to the excess production and deposition of collagen in tissue fibrosis [33, 34]. CHOP signaling has also been suggested to be involved in the inflammation induction by pulmonary cystic fibrosis or myocardial reperfusion injury [9, 35]. We next elucidated whether *CHOP* deficiency contributed to inflammatory cells, including macrophages and neutrophils, infiltration in the UUO kidney. As shown in Figure 6A, F4/80, a macrophage marker, positive cells were detected in the UUO kidneys of WT mice. On the contrary, F4/80 positive stain cells are less frequently detected in the kidneys of *CHOP* deficiency mice. Similarly, Ly6G, the neutrophil marker, is also found to be increased in the UUO kidneys of WT mice on day14 but not in KO mice (Figure 6B). These results indicated the infiltration of macrophages and neutrophils in the kidneys at day 14 after UUO decreased in CHOP deficiency mice.

**Figure 7:**
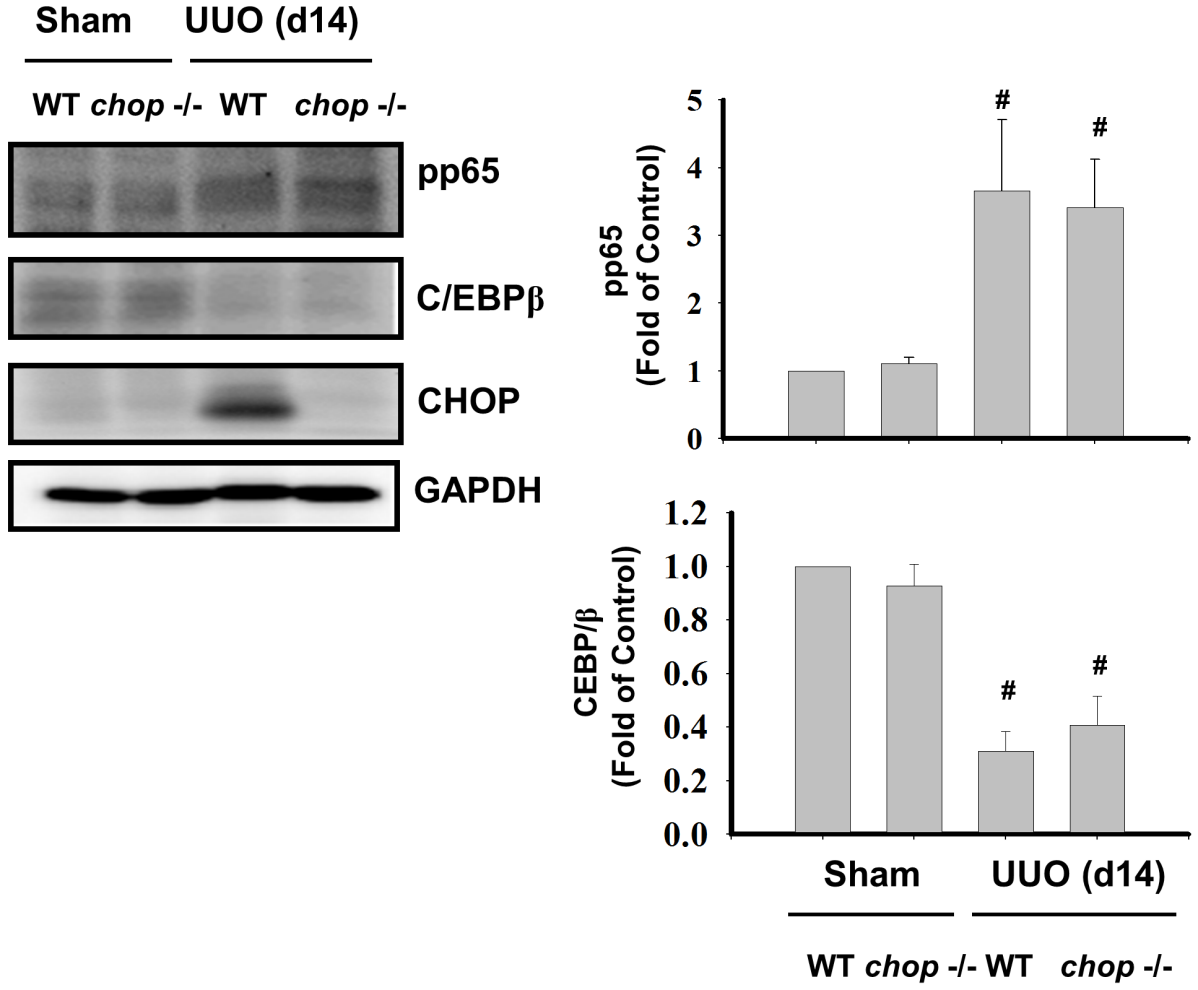
CHOP deficiency does not activate NFκB-p65 phosphorylation and C/EBPβ expression in the kidneys of UUO rats Wild type or *CHOP* knockout micewere surgically administered UUO for 14 days. Sham control was the contralateral normal kidneys. NFκB-p65 phosphorylation and C/EBPβ expression were performed by Western blotting. Data are presented as means ± SEM (n=5/group) for three independent experiments. #*P* < 0.05, vs wild type sham control. WT: Wild type; *CHOP*−/−: CHOP deficiency.

We next ascertained whether *Chop* deficiency contributed to the NF-κB suppression. An increase in phosphorylation of NF-κB-p65 and a decrease in C/EBPβ expression were observed in UUO kidneys of WT mice on day 14, but *Chop* depletion did not activate the NF-κB activation and C/EBPβ expression in UUO kidneys (Figure 7).

## DISCUSSION

CHOP is known as an important apoptosis-induced factor in the ER stress network during numerous disease conditions, including diabetes [18, 36], Parkinson's disease [37], and renal dysfunction [38]. The down-regulation of Bcl-2 has been suggested as one mechanism of CHOP-related apoptosis [39]. Under ER stress, CHOP downregulates the expression of Bcl-2, sensitizing cells to apoptosis, resulting in renal functional and pathological damages in acute kidney injury [22, 40]. JNK activation has been implicated in ER stress-related apoptosis associated with CHOP via regulating the expression and activity of pro- and anti-apoptotic proteins, including Bcl-2 family members [41]. Consistent with our findings, *CHOP* deficiency effectively ameliorates renal cell apoptosis via the inhibition of Bcl-2 down-regulation and JNK activation during obstruction-induced nephropathy. These results suggested that ER stress-induced CHOP/JNK/Bcl-2 signals may contribute chronic renal fibrosis in UUO model.

Cumulating evidence suggested that CHOP was involved in the regulation of inflammatory responses, such as prostaglandin E_2_-stimulated interleukin (IL)-8 production, caspase-11-correlated IL-1β production, and caspase-11-related lipopolysaccharide-induced inflammation [9, 12, 42]. IL-8 is known as a major neutrophil chemo-attractant in various tissues [43–46]. Inflammatory cell infiltration, including macrophages and neutrophils, is one of the main features in the kidney of UUO model [24, 47, 48]. In this study, we found that both Ly6G, a neutrophil marker, and F4/80, a macrophage marker, are markedly increased in the UUO kidneys of wild-type mice, but it was dramatically diminished in *CHOP* knockout mice. These results suggest that CHOP deficiency may have the potential to attenuate inflammatory cells infiltration during the obstructive-induced nephropathy. NF-κB can be activated in the kidneys of UUO animal model and is involved in the tubulointerstitial cellular pro-inflammation and interstitial fibrosis [27, 49, 50]. Over-expression of CHOP can block both cyclosporin A and tacrolimus (FK506)-induced NF-κB activation [51]. CHOP is also found to interact with C/EBPs family proteins like C/EBPβ and then alters the NF-κB activation [16, 52]. These results suggested that CHOP signaling might possess inhibitory potential in NF-κB activation and C/EBPs function. In our experiments, unexpectedly, neither NF-κB activation nor C/EBPβ expression was activated in the UUO kidneys of *CHOP* knockout mice as compared with wild-type mice. These results suggest that the attenuation of inflammation and renal fibrosis during CHOP deficiency may not be correlated to NF-κB or CEBP/β signaling pathway.

Oxidative stress is a crucial feature implicated in renal fibrosis, epithelial-mesenchymal transition (EMT), and inflammation induction [31, 53]. Cumulative evidence indicated that NOX4-based NADPH oxidase is an important factor for oxidative stress induction and pathological alterations in liver and lung fibrosis as well as diabetic nephropathy [54–56]. NOX-4 has been found to be involved in salubrinal (an eIF2α dephosphorylation inhibitor)-enhanced cisplatin-induced oxidative stress and acute nephrotoxicity [57]. ER stress inhibitors can effectively reduce ER stress induction (including CHOP), increased NOX-2/NOX-4 expression, oxidative stress induction, and endothelial cell dysfunction in tunicamycin-treated endothelial cells [58]. Inhibition of ER stress has also been found to reverse oxidative stress induction, NOX-4 expression, and NADPH oxidase activity in diabetic cardiac damage and microvascular dysfunction [59]. Pedruzzi et al. observed that the Nox4 deficiency prevented the UPR markers expression, including CHOP and Bax proteins, and cell death induction in 7-ketocholesterol-treated aortic smooth muscle cells [60]. These findings implied that NOX4 is one of the important oxidative stress signals induced by ER stress, which causes pathological insults. Moreover, overexpression of manganese SOD was capable of attenuating cadmium-induced CHOP protein expression and apoptosis in LLC-PK1 cells; overexpression of catalase did not reduce cadmium-induced CHOP expression, but could inhibit apoptosis [61]. Santos et al. recently suggested that oxidative stress might occur both upstream and downstream of the ER stress response, including the expression of ATF4, Nrf2, and CHOP, and the oxidases like as Nox2/Nox4 may couple ER stress to cellular redox signals, which is alone with the prosurvival or proapoptotic outcome [62]. In this study, we found that lipid peroxidation and NOX-4 expression were markedly increased and SOD1 and catalase were dramatically decreased in UUO kidneys of wild-type mice but not in *CHOP* knockout mice. These results suggest that CHOP is an important factor for the regulation of oxidative stress induction and NOX-4 modulation, which may be involved in the UUO fibrotic process. However, investigation on detailed signaling cascade components is still needed in the future.

Renal fibrogenesis is known to be associated with leukocyte recruitment, angiogenesis, vascular leak, and myofibroblast appearance [63]. The renal myofibroblasts are thought to originate *de novo* in renal fibrosis in which they may represent a stressed and dedifferentiated phenotype of fibroblasts [64]. LeBleu et al. suggested that the source of myofibroblasts in kidney fibrosis included local resident fibroblasts, bone marrow differentiation, endothelial-to-mesenchymal transition, and epithelial-to-mesenchymal transition [65]. Duffield has recently reviewed that the studies in FOXD1-lineage cells provide the evidence that pericytes and resident fibroblasts are the major precursors of myofibroblasts [63]. Moreover, macrophage polarization, including classically-activated M1 proinflammatory macrophages and alternative activation of macrophages (M2), can also regulate the renal fibrosis [66]. Pan et al. recently found that deficiency of M2 macrophages, but not of M1 macrophages, inhibited EMT and renal fibrosis in a mouse UUO model [67]. M2 macrophages have been suggested to be the main source in the progression of renal fibrosis (CKD) following ischemia/reperfusion injury (AKI) [68]. It has been demonstrated that the phenotypic transition of resident renal tubular cells, endothelial cells, and pericytes is involved in the UUO-induced renal fibrosis process [24]. UUO has also been found to do not affect in significant glomerular injury but shows early macrophage infiltration and interstitial fibrosis [25]. Thus, question possibly raised in the future based on the data of current study is CHOP expressed in which cell types are critical in the context of kidney fibrosis.

Recently, Zhang et al. have shown that suppression of Hmgb1/TLR4/NFκB/IL-1β signaling pathway is involved in the CHOP deficiency-prevented UUO-induced renal fibrosis [69]. The present work also demonstrates that CHOP deficiency conspicuously attenuates obstructive-induced renal fibrosis in UUO model. Our novel findings are that CHOP deficiency diminishes not only apoptotic cell death as well as prolonged oxidative stress but also reduces inflammation infiltration in fibrotic process. These findings indicated that CHOP signals may be a potential therapeutic target for the prevention of CKD and ESRD.

## MATERIALS AND METHODS

### Unilateral ureteral obstruction (UUO) mouse model

Mice deficient in CHOP (*CHOP*^−/−^) on a C57BL/6 background were purchased from Jackson Laboratories (Bar Harbor, ME, USA). Adult male *CHOP*^−/−^ mice and WT C57BL/6 mice weighing 20-25 g (6-week-old) were used in this study. The Animal Research Committee of the College of Medicine, National Taiwan University, approved and conducted the study in accordance with the guidelines for the care and use of laboratory animals. The animals were treated humanely and with regard for alleviation of suffering. Mice were housed in a room at a constant temperature of 22±2°C with a 12 h light-dark cycle. The UUO model was performed as described previously [4, 33]. Briefly, the left abdominal incision was made around in the kidney and the ureter was ligated with cotton thread. After suturing, the animals returned to the cage for adapt time point. Contralateral non-obstructed kidneys were served as the control preparations. Mice were sacrificed on 3, 7, and 14 days after UUO induction.

### Histological examination

Renal tissues were isolated and fixed with 10% formaldehyde buffered with PBS, 0.01 M, pH 7.4. Following, the 3-μm-thick sections were prepared. For estimating renal histological injury and renal fibrosis, Masson-trichrome staining sections were used. Renal collagen deposition was as described previously [4].

### Immunohistochemical staining

Three-μm-thick sections were prepared and immunohistochemical staining was performed as described previously [4, 33]. Briefly, the sections were deparaffinized with xylene solution and 75% alcohol for 5 min each. After boiling for 30 min in sodium citrate buffer (PH 6.0), the endogenous peroxidase activity was eliminated with 3% hydrogen peroxide and the non-specific binding reaction was blocked with 5% goat serum for 30 min. The sections were exposed with anti-fibronectin (Cat. No. 610077; BD Biosciences, San Jose, CA, USA), anti-α-smooth muscle actin (αSMA) (Cat. No. A2547; Sigma-Aldrich, St. Louis, MO, USA), anti-Ly6G (Cat. No. 14-5931-82; eBioscience, San Diego, CA, USA) and anti-F4/80 (Cat. No. 14-4801-82; eBioscience) monoclonal antibodies and then incubated with alkaline phosphatase-conjugated goat anti-rabbit or goat anti-mouse IgG. The slides were developed using 1% H_2_O_2_ and DAB in 0.05 M Tris-HCl (pH 7.9), and counterstained with haematoxylin. At least 15 randomly visual fields were sampled from the cortex and medulla for each kidney using a 200 X magnification. Quantification of the results was performed by imageJ software to analyze positive staining.

### Terminal deoxynucleotidyl transferase-mediated dUTP nick-end labeling (TUNEL) fluorescence staining

Renal apoptotic cells were detected by the fluorometric transferase-mediated TUNEL assay (Promega, Madison, WI, USA). The TUNEL stain protocol was follow to the manufacturer instruction. Briefly, renal tissues isolated from UUO or normal mice were fixed in 10% formaldehyde and embedded in paraffin. 3-μm-thick sections were deparaffinized and then rehydration with alcohol. Subsequently, proteinase K was used to digest protein for 20 min and preceded to PBS wash 3 times for 10 min each. Stained the samples with TUNEL fluoresce mixture for 1 h, and Hoechst 33258 (1 μg/ml) counter stain was followed for 15 min. The number of apoptotic cells was counted from 15 random fields under a fluorescence microscopy with 200x magnifications.

### Malondialdehyde (MDA) assay

To assess the oxidative injury in obstruction-induced renal tissues, MDA contents were measured using a MDA detection kit (Cayman, Michigan, USA). Briefly, wild type and CHOP knockout mice were sacrificed and the kidneys were isolated. Renal cortex tissues were homogenized with pestles in 300 μl RIPA buffer contained protease inhibitors, and then centrifuged at 1600 x g for 20 min. The supernatants were added to the reaction mixture for 1 h, and then centrifuged at 1600 x g for 10 min. The fluorescence was read at an excitation wavelength of 530 nm and an emission wavelength of 550 nm in a spectrofluorometer.

### Western blotting

Renal cortex tissues were homogenized and lysed with the RIPA buffer. Whole cell lysates were subsequently centrifuged at 13,000 x g for 30 min and the total protein were collected. Electrophoresis, immunoblotting, and detection were done as described previously [4, 22]. Quantification of the results was performed by densitometric analysis. The relative values of each protein were normalized with GAPDH protein expression. The following primary antibodies were used: CHOP (Cat. No. SC-575), PAI-1 (Cat. No. SC-8979), phospho-N-terminal kinase (pJNK; Cat. No. SC-6254), Jun N-terminal kinase (JNK; Cat. No. SC-1648), and GAPDH (Cat. No. SC-25778) from Santa Cruz (Dallas, Texas, USA); Collagen I (Cat. No. ab34710), Catalase (Cat. No. ab16731), SOD1 (Cat. No. ab13498), NADPH oxidase-4 (NOX-4; Cat. No. ab133303) from Abcam (Cambridge, MA, USA); αSMA (Cat. No. A2547) from Sigma-Aldrich; SOD2 (Cat. No. 13141S) from Cell Signaling (Danvers, MA, USA); Caspase 12 (Cat. No. 3282-100) from Biovision (Milpitas, CA, USA).

### Statistics

The results were obtained in triplicate. Data are expressed as mean ± SEM. All data analysis was performed by one-way analysis of variance (ANOVA) followed by post hoc analysis with Bonferroni's test. P value < 0.05 was considered to indicate a statistically significant difference.
